# Supplementary material for: Influence of Nitrogen Fertilization and Cutting Dynamics on the Yield and Nutritional Composition of White Clover (Trifolium repens L.)
Source: Plants (Basel). 2025 Sep 4;14(17):2765. doi: 10.3390/plants14172765 (PMC12430266; doi:10.3390/plants14172765)
Supplement: Supplementary file 1 [file plants-14-02765-s001.zip › plants-3787394-supplementary.pdf]

Supplementary Materials

Table S1. Evaluation of morphology and performance parameters.

Dose (kg N ha^{-1})	Cutting time (days)	post-cutting evaluation (days)	Leaf length (mm)	Leaf width (mm)	Stem diameter (cm)	Plant Height (cm)	Fresh weight (g)	Dry weight (g)
0	30	7	15.33 ± 0.97 a	25.23 ± 3.10 j-l	0.77 ± 0.15 a	4.10 ± 0.20 u	105.33 ± 9.61 w	18.97 ± 2.42 qr
		14	16.20 ± 1.25 a	31.80 ± 1.65 b-k	1.37 ± 0.15 a	5.57 ± 0.47 q-t	164.00 ± 9.64 r-t	28.87 ± 1.89 op
		21	16.57 ± 0.74 a	31.80 ± 1.65 b-k	1.03 ± 0.06 a	6.83 ± 0.55 m-q	231.67 ± 12.58 q	40.75 ± 1.59 lm
		28	16.73 ± 0.49 a	26.43 ± 0.81 i-l	1.1 0 ± 0.10 a	7.03 ± 0.25 l-q	316.67 ± 22.55 p	44.57 ± 3.15 k-m
		35	17.43 ± 0.85 a	34.17 ± 1.55 a-j	1.17 ± 0.06 a	7.17 ± 0.72 l-q	458.33 ± 46.46 l-o	70.20 ± 6.62 i
		42	17.53 ± 0.97 a	33.47 ± 0.76 b-j	1.23 ± 0.15 a	9.37 ± 0.95 h-l	683.33 ± 35.12 jk	109.93 ± 4.55 h
		49	18.27 ± 0.57 a	30.93 ± 2.00 b-k	1.37 ± 0.06 a	11.30 ± 0.26 f-j	823.33 ± 66.58 g-j	136.57 ± 10.06 e-h
		56	18.87 ± 2.38 a	35.47 ± 2.87 a-i	1.40 ± 0.30 a	12.33 ± 0.31 f-h	850.00 ± 70.00 f-j	113.03 ± 14.42 h
	45	7	14.40 ± 1.59 a	26.87 ± 2.60 h-l	1.03 ± 0.12 a	4.57 ± 0.21 s-u	132.67 ± 8.50 t-w	19.97 ± 1.18 qr
		14	15.37 ± 1.00 a	27.47 ± 3.56 g-l	1.07 ± 0.12 a	6.07 ± 0.71 p-s	188.67 ± 41.43 q-s	30.07 ± 5.63 no
		21	16.00 ± 0.89 a	29.40± 1.74 e-l	1.07 ± 0.23 a	6.40 ± 0.46 o-r	216.00 ± 10.15 q	34.60 ± 1.54 m-o
		28	16.93 ± 0.06 a	29.40 ± 8.70 f-l	1.07 ± 0.06 a	6.90 ± 0.30 m-q	378.33 ± 23.63 n-p	53.17 ± 3.14 jk
		35	16.97 ± 0.49 a	30.40 ± 3.40 c-l	1.10 ± 0.20 a	7.93 ± 0.75 l-p	495.00 ± 13.23 lm	76.37 ± 2.22 i
		42	17.20 ± 1.55 a	31.57 ± 0.49 b-k	1.10 ± 0.10 a	8.97 ± 0.91 i-m	706.67 ± 30.55 ij	114.47 ± 5.35 gh
		49	17.77 ± 1.57 a	33.40 ± 2.02 b-j	1.13 ± 0.06 a	11.00 ± 1.00 g-k	811.67 ± 22.55 g-j	133.30 ± 4.07 e-h
		56	19.43 ± 1.40 a	33.37 ± 4.37 b-j	1.30 ± 0.10 a	12.17 ± 0.76 f-h	866.67 ± 41.63 f-j	120.23 ± 5.54 f-h
	60	7	14.53 ± 0.65 a	22.60 ± 2.17 l	1.00 ± 0.17 a	4.27 ± 0.38 tu	115.67 ± 6.11 uw	20.00 ± 0.82 qr
		14	14.77 ± 1.46 a	32.80 ± 1.15 b-j	1 .00± 0.10 a	6.63 ± 0.35 n-r	143.67 ± 12.01 s-u	23.00 ± 1.76 pq
		21	15.83 ± 0.74 a	32.80 ± 2.01 b-j	1.07 ± 0.06 a	6.93 ± 0.99 m-q	233.67 ± 17.04 q	37.41 ± 2.37 l-n
		28	16.07 ± 1.18 a	28.57 ± 0.38 f-l	1.07 ± 0.12 a	7.10 ± 0.26 l-q	331.00 ± 31.19 p	46.83 ± 4.24 kl
		35	16.90 ± 1.14 a	27.77 ± 2.34 g-l	1.07 ± 0.15 a	7.93 ± 0.46 l-p	518.33 ± 53.46 lm	79.77 ± 8.27 i
		42	15.37 ± 0.71 a	36.57 ± 0.86 a-g	1.07 ± 0.06 a	8.50 ± 0.95 j-o	721.67 ± 55.30 ij	116.63 ± 9.28 f-h
		49	17.87 ± 0.99 a	32.53 ± 1.80 b-k	1.20 ± 0.10 a	11.17 ± 1.04 g-j	793.33 ± 73.71 h-j	131.10 ± 12.00 e-h
		56	18.33 ± 2.58 a	36.3 ± 2.98 a-h	1.30 ± 0.10 a	12.83 ± 0.29 e-g	906.67 ± 37.53 e-i	125.43 ± 6.16 f-h
60	30	7	14.83 ± 1.46 a	28.10 ± 1.15 f-l	0.97 ± 0.15 a	5.03 ± 0.15 r-u	130.33 ± 4.73 t-w	19.37 ± 0.86 qr
		14	13.67 ± 1.95 a	24.03 ± 0.90 kl	1.03 ± 0.06 a	6.90 ± 0.70 m-q	196.67 ± 8.08 qr	35.20 ± 1.82 m-o
		21	16.80 ± 0.72 a	24.03 ± 0.90 kl	1.00 ± 0.00 a	7.17 ± 0.25 l-q	355.00 ± 18.03 op	63.56 ± 4.37 ij
		28	17.60 ± 0.82 a	30.10 ± 0.85 c-l	1.20 ± 0.00 a	8.77 ± 0.47 i-n	490.00 ± 20.00 l-n	64.97 ± 2.40 ij
		35	17.87 ± 0.45 a	40.03 ± 7.46 a-e	1.23 ± 0.15 a	11.3 ± 1.82 f-j	710.00 ± 22.91 ij	105.97 ± 2.97 h
		42	20.37 ± 1.29 a	37.00 ± 0.70 a-g	1.23 ± 0.12 a	15.07 ± 2.31 d-f	988.33 ± 23.63 e-h	150.27 ± 3.01 d-f
		49	20.80 ± 0.46 a	40.93 ± 0.84 a-c	1.23 ± 0.06 a	16.67 ± 0.58 c-e	1041.67 ± 58.38 d-g	163.67 ± 13.53 c-e
		56	23.23 ± 0.74 a	46.30 ± 3.40 a	1.30 ± 0.10 a	23.00 ± 4.00 ab	1356.67 ± 100.17 a-c	224.27 ± 19.05 ab
	45	7	14.60 ± 0.75 a	24.07 ± 0.97 kl	0.87 ± 0.25 a	4.33 ± 0.15 tu	108.33 ± 4.04 w	17.33 ± 0.50 r
		14	14.47 ± 1.01 a	27.53 ± 3.46 g-l	0.90 ± 0.10 a	6.87 ± 0.81 m-q	182.33 ± 32.72 q-s	31.60 ± 3.56 no
		21	17.27 ± 0.75 a	27.53 ± 2.42 g-l	1.13 ± 0.06 a	8.57 ± 0.64 j-n	401.00 ± 23.52 m-p	70.18 ± 8.27 i
		28	20.50 ± 1.65 a	31.33 ± 2.53 b-k	1.17 ± 0.12 a	8.80 ± 0.82 i-n	536.67 ± 18.93 kl	71.33 ± 2.55 i
		35	20.57 ± 0.93 a	39.73 ± 3.43 a-e	1.30 ± 0.10 a	11.3 ± 1.35 f-j	846.67 ± 60.28 f-j	126.03 ± 8.10 f-h
		42	21.20 ± 1.15 a	38.13 ± 0.75 a-f	1.33 ± 0.06 a	14.93 ± 0.64 d-f	1088.33 ± 46.46 c-f	168.30 ± 8.71 c-e
		49	21.97 ± 0.85 a	40.50 ± 0.56 a-c	1.33 ± 0.32 a	18.83 ± 1.89 a-d	1140.00 ± 101.49 b-e	177.03 ± 15.24 b-d
		56	23.27 ± 1.01 a	46.40 ± 0.79 a	1.40 ± 0.20 a	24.67 ± 3.51 a	1606.67 ± 135.77 a	262.30 ± 20.92 a
	60	7	15.77 ± 1.29 a	29.40 ± 1.95 e-l	0.71 ± 0.51 a	5.10 ± 0.20 r-u	131.00 ± 13.45 t-w	20.07 ± 2.33 qr
		14	16.27 ± 2.08 a	30.27 ± 7.18 d-l	0.87 ± 0.21 a	7.13 ± 0.49 l-q	228.00 ± 14.42 q	36.73 ± 0.55 l-o
		21	17.40 ± 1.47 a	30.27 ± 2.10 c-l	0.97 ± 0.29 a	8.33 ± 0.81 k-o	400.00 ± 32.79 m-p	64.47 ± 4.18 ij
		28	19.60 ± 1.28 a	28.50 ± 0.80 f-l	1.10 ± 0.10 a	8.77 ± 0.31 i-n	535.00 ± 32.79 kl	70.63 ± 4.57 i
		35	20.20 ± 2.46 a	38.10 ± 1.35 a-f	1.13 ± 0.12 a	11.57 ± 0.96 f-i	803.33 ± 35.12 g-j	117.63 ± 5.15 f-h
		42	22.47 ± 0.86 a	40.40 ± 0.70 a-d	1.17 ± 0.12 a	17.40 ± 0.6 b-d	945.00 ± 49.24 e-h	146.10 ± 7.24 d-g
		49	23.23 ± 1.21 a	41.73 ± 1.05 ab	1.16 ± 0.15 a	19.00 ± 1.00 a-d	1298.33 ± 22.55 a-d	202.53 ± 3.08 a-c
		56	24.23 ± 1.22 a	37.83 ± 1.76 a-f	1.27 ± 0.06 a	20.67 ± 0.58 a-c	1446.67 ± 155.03 ab	236.37 ± 26.78 a

Table S2. Evaluation of nutritional composition parameters

Dose (kg N ha^{-1})	Cutting time (days)	time evaluation	%Protein	%ADF	%NDF	Gross energy (Kcal)	%in vitro digestibility of dry matter	%Ash	%Crude fiber	%moisture
**0**	**30**	**1**	24.04 ± 0.35 de	22.41 ± 0.64 d	25.75 ± 2.00 cd	4754.71 ± 28.51 ab	93.88 ± 1.15 a	10.78 ± 0.53 b-d	10.24 ± 1.13 a	8.17 ± 0.32 a
		**2**	25.82 ± 1.06 b-d	22.59 ± 0.43 d	26.82 ± 0.20 a-d	4475.25 ± 5.00 ef	80.57 ± 0.89 b	10.59 ± 0.24 cd	14.90 ± 0.72 a	8.46 ± 0.45 a
		**3**	27.23 ± 0.84 a-c	25.14 ± 0.44 a-d	24.35 ± 1.23 cd	4523.2 ± 20.99 d-f	80.05 ± 0.64 b	10.22 ± 0.92 d	16.05 ± 0.45 a	8.46 ± 0.45 a
	**45**	**1**	28.05 ± 0.35 ab	26.97 ± 1.18 a	27.15 ± 1.43 a-d	4817.52 ± 17.07 a	93.81 ± 0.21 a	12.34 ± 0.50 ab	10.90 ± 0.96 a	8.28 ± 0.45 a
		**2**	26.19 ± 0.34 a-d	22.79 ± 0.47 d	24.47 ± 1.09 cd	4500.4 ± 7.77 d-f	80.25 ± 0.93 b	9.99 ± 0.15 d	16.11 ± 0.22 a	9.59 ± 1.02 a
		**3**	27.71 ± 0.98 a-c	24.01 ± 1.16 b-d	24.94 ± 0.66 cd	4615.63 ± 24.34 cd	80.21 ± 0.58 b	10.56 ± 0.22 cd	17.22 ± 0.96 a	9.59 ± 1.02 a
	**60**	**1**	26.52 ± 0.74 a-c	24.31 ± 1.46 a-d	26.06 ± 0.82 cd	4694.55 ± 60.86 bc	92.33 ± 0.21 a	12.05 ± 0.48 a-c	10.60 ± 1.42 a	7.42 ± 0.54 a
		**2**	21.94 ± 0.48 ef	26.09 ± 0.85 a-c	30.63 ± 2.43 a	4450.6 ± 41.82 ef	76.34 ± 1.23 d-f	10.94 ± 0.73 a-d	18.68 ± 0.57 a	8.36 ± 0.78 a
		**3**	27.30 ± 0.25 a-c	24.93 ± 0.66 a-d	30.38 ± 1.20 ab	4548.68 ± 32.69 de	79.89 ± 0.63 bc	10.56 ± 0.70 cd	15.85 ± 0.61 a	8.36 ± 0.78 a
**60**	**30**	**1**	28.46 ± 0.65 a	24.25 ± 0.83 a-d	25.84 ± 1.63 cd	4520 ± 54.19 d-f	92.93 ± 0.87 a	12.52 ± 0.23 a	12.15 ± 0.87 a	6.35 ± 0.42 a
		**2**	22.67 ± 0.50 e	26.17 ± 0.90 a-c	30.54 ± 0.44 a	4413.38 ± 56.00 f	75.93 ± 0.85 d-f	11.23 ± 0.60 a-d	19.28 ± 0.57 a	7.49 ± 0.45 a
		**3**	28.44 ± 0.51 a	24.86 ± 1.14 a-d	23.60 ± 0.91 d	4522.33 ± 32.57 d-f	79.4 ± 0.70 bc	10.74 ± 0.49 b-d	19.03 ± 0.98 a	7.49 ± 0.45 a
	**45**	**1**	25.47 ± 0.87 cd	23.73 ± 0.79 cd	26.12 ± 1.08 b-d	4479.81 ± 41.03 ef	93.24 ± 0.65 a	10.96 ± 0.29 a-d	9.88 ± 0.51 a	6.97 ± 0.88 a
		**2**	19.80 ± 0.77 f	26.08 ± 1.12 a-c	28.55 ± 1.81 a-c	4494.94 ± 67.99 ef	75.54 ± 1.31 ef	10.55 ± 1.82 d	19.00 ± 0.72 a	9.11 ± 1.33 a
		**3**	25.40 ± 0.49 cd	24.92 ± 0.50 a-d	27.10 ± 1.09 a-d	4480.81 ± 45.76 ef	78.35 ± 1.18 b-d	11.54 ± 1.22 a-d	18.35 ± 1.28 a	8.77 ± 1.19 a
	**60**	**1**	25.74 ± 0.93 b-d	23.25 ± 1.65 d	27.65 ± 0.74 a-d	4487.17 ± 40.51 ef	92.8 ± 0.40 a	10.52 ± 1.17 cd	11.13 ± 0.63 a	6.35 ± 0.44 a
		**2**	19.89 ± 1.03 f	26.61 ± 0.94 ab	27.72 ± 1.49 a-d	4482.66 ± 15.64 ef	74.12 ± 0.81 f	10.08 ± 0.57 d	21.86 ± 0.26 a	8.17 ± 0.21 a
		**3**	25.41 ± 1.13 cd	26.65 ± 0.98 ab	28.07 ± 2.13 a-c	4470.64 ± 57.55 ef	77.35 ± 0.94 c-e	10.66 ± 0.27 cd	19.01 ± 0.66 a	8.19 ± 0.22 a

Table S3. Equations of the most suitable models for each treatment in morphology.

Treatments	Leaf width	Stem diameter	Plant height	Leaf length
**0 kg N ha^{-1} (cut at 30 days)**	y = 26.2295 * exp (0.0054 * x)	y = 0.9359 - 0.3342spline_1(x) - 0.0672spline_2(x) + 0.1742spline_3(x) + 0.2936spline_4(x) + 0.3824spline_5(x) + 0.4871spline_6(x)	y =7.0368 - 5.0136spline_1(x) - 2.7657spline_2(x) - 0.5458spline_3(x) + 1.9620spline_4(x) + 5.0945spline_5(x) + 8.3053spline_6(x)	y = 14.6842 - 0.2718spline_1(x) + 0.8341spline_2(x) + 1.9023spline_3(x) + 2.9347spline_4(x) + 4.0626spline_5(x) + 5.2223spline_6(x)
**0 kg N ha^{-1} (cut at 45 days)**	y = 26.0870 * exp (0.0046 * x)	y =0.9750+ 0.0676spline_1(x) + 0.0724spline_2(x) + 0.0830spline_3(x) + 0.1357spline_4(x) + 0.2448spline_5(x) + 0.3715spline_6(x) +	y =7.0965 - 4.3785spline_1(x) - 2.4480spline_2(x) - 0.5104spline_3(x) + 1.8348spline_4(x) + 4.7793spline_5(x) + 7.8192spline_6(x)	y =14.3785 - 1.2679spline_1(x) + 0.2564spline_2(x) + 1.7238spline_3(x) + 3.0185spline_4(x) + 4.5159spline_5(x) + 6.1317spline_6(x)
**0 kg N ha^{-1} (cut at 60 days)**	y = 24.5390 * exp (0.0074 * x)	y = 0.9548 + 0.0036spline_1(x) + 0.0435spline_2(x) + 0.0862spline_3(x) + 0.1633spline_4(x) + 0.2745spline_5(x) + 0.3837spline_6(x)	y = 7.2791 - 4.5631spline_1(x) - 2.4953spline_2(x) - 0.5150spline_3(x) + 1.7402spline_4(x) + 4.8866spline_5(x) + 8.2258spline_6(x)	y = 14.0866 - 1.0371spline_1(x) + 0.3511spline_2(x) + 1.7515spline_3(x) + 3.1108spline_4(x) + 4.3501spline_5(x) + 5.5603spline_6(x)
**60 kg N ha^{-1} (cut at 30 days)**	y = 21.6224 * exp (0.0135 * x)	y =0.9650 - 0.1039spline_1(x) + 0.0203spline_2(x) + 0.1534spline_3(x) + 0.2600spline_4(x) + 0.3016spline_5(x) + 0.3336spline_6(x)	y = 10.9634 - 10.2167spline_1(x) - 6.6564spline_2(x) - 2.8573spline_3(x) + 2.6495spline_4(x) + 10.0782spline_5(x) + 17.9661spline_6(x)	y = 13.0444 * exp (0.0101 * x)
**60 kg N ha^{-1} (cut at 45 days)**	y = 22.4397 * exp (0.0129 * x)	y = 0.9788 - 0.3459spline_1(x) - 0.0980spline_2(x) + 0.1491spline_3(x) + 0.3321spline_4(x) + 0.4287spline_5(x) + 0.5128spline_6(x)	y = 11.5206 - 11.7274spline_1(x) - 7.5319spline_2(x) - 3.1952spline_3(x) + 2.7838spline_4(x) + 11.1539spline_5(x) + 20.0374spline_6(x)	y = 16.1507 - 5.6457spline_1(x) - 1.8884spline_2(x) + 1.8888spline_3(x) + 4.9558spline_4(x) + 7.2882spline_5(x) + 9.5519spline_6(x)
**60 kg N ha^{-1} (cut at 60 days)**	y = 29.5885- 4.8922spline_1(x) - 1.4354spline_2(x) + 2.3738spline_3(x) + 7.5748spline_4(x) + 11.4213spline_5(x) + 14.5463spline_6(x)	y = 0.8723 - 0.2311spline_1(x) - 0.0396spline_2(x) + 0.1550spline_3(x) + 0.2872spline_4(x) + 0.3331spline_5(x) + 0.3678spline_6(x)	y =10.9720 - 10.6198spline_1(x) - 6.4348spline_2(x) - 2.0658spline_3(x) + 3.6387spline_4(x) + 10.0499spline_5(x) + 16.4038spline_6(x)	y = 17.0687 - 4.6073spline_1(x) - 1.7323spline_2(x) + 1.2392spline_3(x) + 4.3903spline_4(x) + 7.4105spline_5(x) + 10.3684spline_6(x)

Table S4. Equations of the most suitable models for each treatment in yield

Treatments	Fresh weight	Dry weight
**0 kg N ha^{-1} (cut at 30 days)**	y = 407.3601 - 567.4168spline_1(x) - 348.7562spline_2(x) - 117.4618spline_3(x) + 173.8034spline_4(x) + 482.7867spline_5(x) + 784.4048spline_6(x)	y = 61.4294 - 81.7544spline_1(x) - 48.7482spline_2(x) - 14.0189spline_3(x) + 29.1107spline_4(x) + 69.8870spline_5(x) + 106.9531spline_6(x)
**0 kg N ha^{-1} (cut at 45 days)**	y = 421.4597- 559.7793spline_1(x) - 339.6552spline_2(x) - 105.8107spline_3(x) + 182.1549spline_4(x) + 477.7884spline_5(x) + 766.7616spline_6(x)	y = 63.2510 - 85.3882spline_1(x) - 50.6440spline_2(x) - 13.8779spline_3(x) + 30.8267spline_4(x) + 72.1301spline_5(x) + 110.2044spline_6(x)
**0 kg N ha^{-1} (cut at 60 days)**	y =420.0780 - 597.1981spline_1(x) - 365.3591spline_2(x) - 118.2696spline_3(x) + 186.1826spline_4(x) + 501.4890spline_5(x) + 813.2333spline_6(x)	y = 63.4949 - 88.8570spline_1(x) - 53.5758spline_2(x) - 15.7867spline_3(x) + 30.9658spline_4(x) + 74.8502spline_5(x) + 115.8983spline_6(x)
**60 kg N ha^{-1} (cut at 30 days)**	y =592.1166 - 866.1552spline_1(x) - 526.6929spline_2(x) - 171.1306spline_3(x) + 251.2700spline_4(x) + 715.4544spline_5(x) + 1189.3709spline_6(x)	y =95.6177 - 133.9321spline_1(x) - 83.9929spline_2(x) - 31.9432spline_3(x) + 33.9399spline_4(x) + 114.0672spline_5(x) + 197.4789spline_6(x)
**60 kg N ha^{-1} (cut at 45 days)**	y = 666.3088 - 1037.2490spline_1(x) - 629.8206spline_2(x) - 205.1848spline_3(x) + 288.1657spline_4(x) + 839.4017spline_5(x) + 1410.9958spline_6(x)	y = 106.9319 - 159.3226spline_1(x) - 99.6650spline_2(x) - 37.4575spline_3(x) + 39.4794spline_4(x) + 132.8454spline_5(x) + 231.0521spline_6(x)
**60 kg N ha^{-1} (cut at 60 days)**	y = 651.6571 - 955.1303spline_1(x) - 580.5190spline_2(x) - 190.6034spline_3(x) + 271.8011spline_4(x) + 791.0229spline_5(x) + 1315.0859spline_6(x)	y = 103.7493 - 147.5651spline_1(x) - 93.3096spline_2(x) - 36.4156spline_3(x) + 36.9088spline_4(x) + 126.2810spline_5(x) + 217.8498spline_6(x

Table S5. Spline values in generalized additive models (GAM)

post-cutting evaluation (days)	spline_1	spline_2	spline_3	spline_4	spline_5	spline_6
7	0.1667	0.6667	0.1667	0	0	0
14	0.0311	0.5224	0.4334	0.0131	0	0
21	0.0005	0.2468	0.6477	0.105	0	0
28	0	0.0607	0.5967	0.3387	0.0039	0
35	0	0.0039	0.3387	0.5967	0.0607	0
42	0	0	0.105	0.6477	0.2468	0.0005
49	0	0	0.0131	0.4334	0.5224	0.0311
56	0	0	0	0.1667	0.6667	0.1667
